# In Vitro Differentiation Potential of Human Placenta Derived Cells into Skin Cells

**DOI:** 10.1155/2015/841062

**Published:** 2015-07-01

**Authors:** Ruhma Mahmood, Mahmood S. Choudhery, Azra Mehmood, Shaheen N. Khan, Sheikh Riazuddin

**Affiliations:** ^1^National Center of Excellence in Molecular Biology, University of the Punjab, Lahore, Pakistan; ^2^University of Health Sciences, Lahore, Pakistan; ^3^Tissue Engineering and Regenerative Medicine Laboratory, King Edward Medical University, Lahore, Pakistan; ^4^Allama Iqbal Medical College, Lahore, Pakistan

## Abstract

Skin autografting is the most viable and aesthetic technique for treatment of extensive burns; however, this practice has potential limitations. Harvesting cells from neonatal sources (such as placental tissue) is a simple, inexpensive, and noninvasive procedure. In the current study authors sought to evaluate in vitro potential of human placenta derived stem cells to develop into skin-like cells. After extensive washing, amniotic membrane and umbilical cord tissue were separated to harvest amniotic epithelial cells (AECs) and umbilical cord mesenchymal stem cells (UC-MSCs), respectively. Both types of cells were characterized for the expression of embryonic lineage markers and their growth characteristics were determined. AECs and UC-MSCs were induced to differentiate into keratinocytes-like and dermal fibroblasts-like cells, respectively. After induction, morphological changes were detected by microscopy. The differentiation potential was further assessed using immunostaining and RT-PCR analyses. AECs were positive for cytokeratins and E-Cadherin while UC-MSCs were positive for fibroblast specific makers. AECs differentiated into keratinocytes-like cells showed positive expression of keratinocyte specific cytokeratins, involucrin, and loricrin. UC-MSCs differentiated into dermal fibroblast-like cells indicated expression of collagen type 3, desmin, FGF-7, fibroblast activation protein alpha, procollagen-1, and vimentin. In conclusion, placenta is a potential source of cells to develop into skin-like cells.

## 1. Introduction

Deep burn injuries such as full-thickness wounds require restoration of both the epidermal and the dermal layers of the skin. In normal wound healing, migration and proliferation of keratinocytes from the wound edges result in reepithelization [1] while dermis restoration is dependent on growth factor secretion by macrophages, platelets, and fibroblasts and by fibroblast proliferation [2]. These processes are defective in patients with full-thickness burn injuries due to damage to both layers. Although skin autografting is the most viable and aesthetic technique for the treatment of such injuries, this practice has potential limitations that exposes the donor to additional wounds. Stem cells present in various adult and neonatal tissues show huge potential for tissue engineering and regenerative medicine applications. Harvesting cells from placental tissue is advantageous as it poses no risk to donor, is noninvasive procedure, and is a readily available cell source. Although cord tissue and amniotic membrane of placenta have been recognized as a promising source of stem cells, their therapeutic potential in wound healing has not yet been widely investigated. In the present study, we have evaluated the in vitro differentiation potential of stem cells isolated from cord tissue and amniotic membrane of placenta into skin-like cells, that is, keratinocytes and fibroblasts.

In USA alone, there are 500,000 burn injuries per year due to which approximately 3500 deaths occur annually and this ratio is even higher in developing countries [3]. In Pakistan, burns are the second leading cause of disability and the 11th leading cause of premature deaths [4]. These facts thus necessitate an urgent need for the identification of cell sources that are plentiful and safe and with no ethical issues. Human placenta is one such source that can provide cells for therapeutic strategies. Additionally, immunomodulatory and immunosuppressive properties of placenta derived cells make it an ideal option for use in allogeneic therapy.

In the current study amniotic membrane and umbilical cord tissue were isolated and processed to obtain AECs and UC-MSCs, respectively. Both types of cells were characterized for cell surface and embryonic lineage markers. Further, growth characteristics were determined by analyzing plating efficiency and number and time of population doubling. AECs and UC-MSCs were induced to differentiate in vitro into keratinocytes and fibroblasts, respectively. Differentiated cells displayed significant morphological alterations. Differentiation into keratinocytes- and fibroblast-like cells was further confirmed using immunostaining and reverse transcriptase polymerase chain reaction (RT-PCR). The results of current study suggest that pure populations of AECs and UC-MSCs isolated from human placenta are capable of in vitro differentiation into functional skin cells and thus can be implied for regenerating damaged tissue.

## 2. Materials and Methods

### 2.1. Collection of Placenta

Human placentas (*n* = 15) for the isolation of AECs and UC-MSCs were obtained from the maternity hospitals after full-term, uncomplicated elective cesarean deliveries. All samples were collected from donors who were negative for HBV, HCV, and HIV. The umbilical cord tissue and amniotic membrane were separated in sterilized conditions and processed within two hours. Informed consent was obtained from each donor prior to collection. All protocols used in this study were approved by the institution review board (IRB) at the National Centre of Excellence in Molecular Biology (CEMB), University of the Punjab, Lahore, Pakistan. The reagents used in the study were purchased from Sigma Aldrich, USA; Invitrogen, USA; Abcam, UK and Santa Cruz Biotechnology USA. A complete list of reagents and antibodies have been given in Table 2.

### 2.2. Isolation of Cells

#### 2.2.1. Amniotic Epithelial Cells (AECs)

AECs from amniotic membrane tissue were obtained by direct explant technique [5]. Briefly, amniotic membrane was peeled off from chorion and washed several times with sterile phosphate buffered saline (PBS, Sigma Aldrich, USA). Approximately 1 cm^2^ pieces of amniotic membrane were explanted in 25 cm^2^ culture flask in AECs expansion medium, that is, DMEM supplemented with 10% FCS, 100 U/mL penicillin, and 100 mg/mL streptomycin (Sigma Aldrich, USA) and then incubated in a humidified incubator at 37°C and 5% CO_2_. Medium was first replaced after 48 hours without disturbing the tissue pieces and after every 3 days thereafter for 10–14 days. Tissue pieces were removed and cells were harvested using 0.25% trypsin-EDTA (Sigma Aldrich, USA) and counted with hemocytometer and 75,000 cells were replated in 75 cm^2^ culture flasks. Passage 2 cells were then characterized by plating 1 × 10^4^ cells per well in a multiwall plate and were incubated with primary antibodies for CD45, CK8, CK18, CK19 (Abcam, UK), and E-Cadherin (Santa Cruz Biotechnology, USA) overnight at 4°C. The cells were washed with PBS and then incubated with respective secondary antibodies at room temperature.

#### 2.2.2. Umbilical Cord Mesenchymal Stem Cells (UC-MSCs)

UC-MSCs were isolated using a nonenzymatic digestion technique as described [6]. Briefly 5-inch cord tissue piece was extensively washed with PBS containing 100 U/mL penicillin and 100 mg/mL streptomycin (Sigma Aldrich, USA). Tissue piece was cut and minced to obtain small fine pieces that were then placed in a 25 cm^2^ culture flask (Corning Inc., USA). 5 mL of UC-MSCs expansion medium (DMEM supplemented with 10% FBS, 100 U/mL penicillin, and 100 mg/mL streptomycin (Sigma Aldrich, USA)) was added in the culture flask and tissue pieces were evenly distributed. The culture flasks were incubated at 37°C and 5% CO_2_ in humidified incubator. Cell outgrowth was observed 3-4 days after culturing and tissue pieces were removed after 8–10 days. Cell colonies appeared in the culture within 2 weeks. The cells were harvested using trypsin-EDTA and 75,000 cells were replated in new 75 cm^2^ culture flasks. At passage 2, these cells were incubated with primary antibodies for CD45, CD49, CD73, and CD90 (Abcam, UK) overnight at 4°C. Primary antibodies were removed and cells were incubated with secondary antibodies at room temperature.

### 2.3. Growth Characteristics

#### 2.3.1. Plating Efficiency

Plating efficiency was determined as reported previously [7]. Briefly each type of cells at second passage was seeded in 25 cm^2^ culture flasks at a concentration of 50 cells per cm^2^. Cultures were fed with fresh medium after every 3 days till 2 weeks, after which the cells were fixed with methanol and stained with crystal violet dye (Sigma Aldrich, USA) overnight at room temperature. The resultant colonies were counted and percentage plating efficiency was calculated by using following formula:

(1) %  plating efficiency=colonies countednumber of cells seeded×100.

#### 2.3.2. Number and Time of Population Doublings

Both AECs and UC-MSCs were serially passaged under standard culture conditions for analysis of cumulative population doublings and doubling time. Briefly, at first passage, 1 × 10^5^ cells were counted and plated in a 25 cm^2^ culture flask. At 80%–90% confluency, cells were subcultured, counted, and plated at the same density as described above. This procedure was repeated for several passages until the cells fail to reach 90% confluency even after 3 weeks [7]. Following formulae were used to determine cumulative population doublings and doubling time [6]:

(2) cPDs=log⁡NNo×3.3,DT=CTcPDs,

where cPDs represents cumulative population doublings, *No* is the number of cells plated and *N* is the number of cells harvested, DT is doubling time, and CT is total time.

### 2.4. Differentiation Assays

#### 2.4.1. Differentiation of Amniotic Epithelial Cells into Keratinocyte-Like Cells

AECs were obtained as described above and 1 × 10^4^ cells at passage 2 were plated in 6-well plates in expansion medium. When cells attain their normal morphology, differentiation process was initiated by replacing expansion medium with keratinocyte differentiation medium (DMEM + HAM F12 (3 : 1), Sigma Aldrich, USA) supplemented with 10% FCS, 100 IU/mL penicillin, 100 mg/mL streptomycin, 0.5 mg/mL hydrocortisone, 1% insulin transferrin (Sigma Aldrich, USA), and 15 ng/mL keratinocytes growth factor (Invitrogen, USA). Keratinocytes differentiation medium was replaced twice a week for 15 days. Cells grown in AECs expansion medium for similar time points served as control. After 15 days, differentiation of epithelial cells into keratinocytes-like cells was assessed using immunohistochemistry and RT-PCR analysis.

#### 2.4.2. Differentiation of UC-MSCs into Dermal Fibroblast-Like Cells

UC-MSCs isolated from cord tissue were induced to differentiate into fibroblast-like cells at passage 2. Briefly, 1 × 10^4^ UC-MSCs were plated in each well of a 6-well plate in complete expansion medium. When cells attain their normal morphology, the expansion medium was replaced with fibroblast differentiation medium (DMEM supplemented with 10% FCS (Sigma Aldrich, USA)), 100 IU/mL penicillin, 100 mg/mL streptomycin, 5 ug/mL insulin, and 1 ng/mL basic fibroblast growth factor (Sigma Aldrich, USA). The medium was replaced twice a week for 15 days. Cells grown in expansion medium served as control. Morphological changes were observed during induction period. Further, immunostaining and RT-PCR were performed to assess differentiation into dermal fibroblast-like cells.

### 2.5. Assessment of Differentiated Keratinocytes and Dermal Fibroblast-Like Cells

#### 2.5.1. Immunostaining

Immunostaining was performed for the expression of keratinocytes specific markers: CK5, CK10, involucrin (Sigma Aldrich, USA), and loricrin (Abcam, UK) and dermal fibroblast specific markers, collagen-3 (Santa Cruz Biotechnology, USA), desmin (Santa Cruz Biotechnology, USA), FAP-*α* (Abcam, UK), and procollagen-1 (Santa Cruz Biotechnology, USA). Briefly, cells were washed with PBS three times and treated with 4% paraformaldehyde (PFA) for 15 minutes. The fixed cells were washed with PBS (5 × 3 times) and incubated with the primary antibodies overnight at 4°C. Incubation at room temperature with respective secondary antibodies (1 : 200) was performed for 1 hour at 37°C. After washing with PBS, cell nuclei were stained with DAPI (Sigma Aldrich, USA) and observed microscopically.

#### 2.5.2. Reverse Transcriptase Polymerase Chain Reaction (RT-PCR)

Expression of lineage specific genes was carried out by RT-PCR. Briefly, cells were cultured for 15 days in the respective differentiation medium and total RNA was extracted using Trizol reagent (Sigma Aldrich, USA). RNA was quantified with a ND-1000 spectrophotometer (NanoDrop Technologies, USA). 1.5 ug of RNA sample was used for cDNA synthesis using MMLV (Moloney murine leukaemia virus) reverse transcriptase kit (Invitrogen, USA). The following PCR conditions were used: 94°C for 5 minutes followed by 35 cycles for 45 sec at 94°C, 45 sec at respective annealing temperature (mentioned against each gene), and 72°C for 45 sec. RT-PCR for cells differentiated into keratinocytes-like cells was done for the expression of CK1 (57°C), CK10 (57°C), CK14 (58°C), and E-Cad. (57°C) while for cells differentiated into dermal fibroblast-like cells it was done using collagen-3 (59°C), desmin (58°C), FGF7 (58°C), and vimentin (57°C). Beta actin was used as an internal control. Sequences for the primer pairs and their product lengths (bp) are given in Table 1. Gel bands were quantified with image J software (http://rsbweb.nih.gov/ij/).

### 2.6. Statistical Analysis

Statistical analysis of data was performed using Graphpad prism 5 software. The data was presented as mean ± standard deviation. Unpaired *t*-test was performed to compare two groups. *P* values ≤ 0.05 were considered to be statistically significant.

## 3. Results

### 3.1. Biological Properties of AECs and UC-MSCs

**Figure 1:**
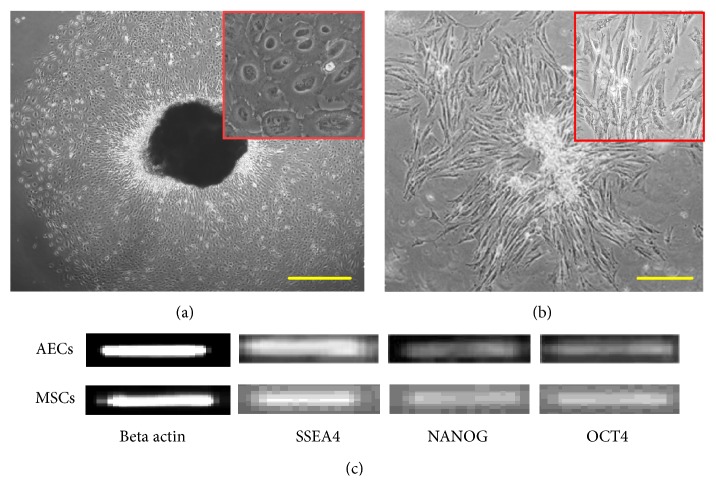
Morphological appearance of human amniotic epithelial cells (AECs) and umbilical cord mesenchymal stem cells (UC-MSCs). Cells in the primary culture of AECs (a) and UC-MSCs (b). AECs displayed rounded shape while UC-MSCs showed spindle shaped morphology. Insets show morphological features at higher magnification. Both types of cells showed expression of embryonic lineage markers SSEA4, NANOG, and OCT4 (c). AECs: amniotic epithelial cells, UC-MSCs: mesenchymal stem cells, SSEA4: stage specific embryonic antigen 4, and OCT4: octamer binding protein 4.

Cells from amniotic membrane and cord tissue were successfully isolated. Cell outgrowth from both types of tissues was observed in 3-4 days after culturing. Epithelial cells were round in shape (Figure 1(a)) while UC-MSCs showed spindle shaped morphology (Figure 1(b)). Both types of cells were plastic adherent and grew into colonies; however, colonies of AECs were more like a sheet of cells. The neonatal origin of both sources was confirmed by RT-PCR using NANOG, OCT4, and SSEA4 (Figure 1(c)). The results of the current study are in line with previous studies that show similar findings [8–11].

### 3.2. Characterization of Cells

**Figure 2:**
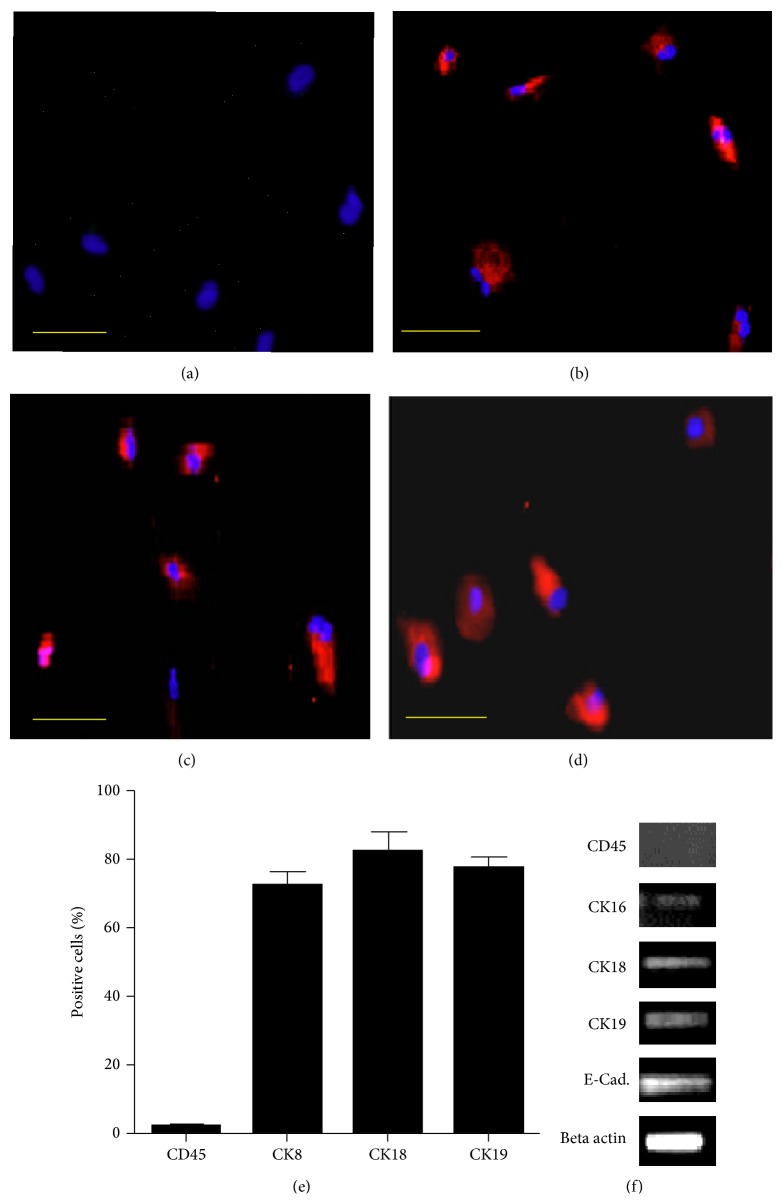
Immunostaining for AECs; (a) CD 45 negative. (b)–(d) CK8, CK18, and CK19 positive. (e) shows percentage of positive cells. RT-PCR for AECs; (f) CK16, CK18, CK19, and E-Cadherin positive.

**Figure 3:**
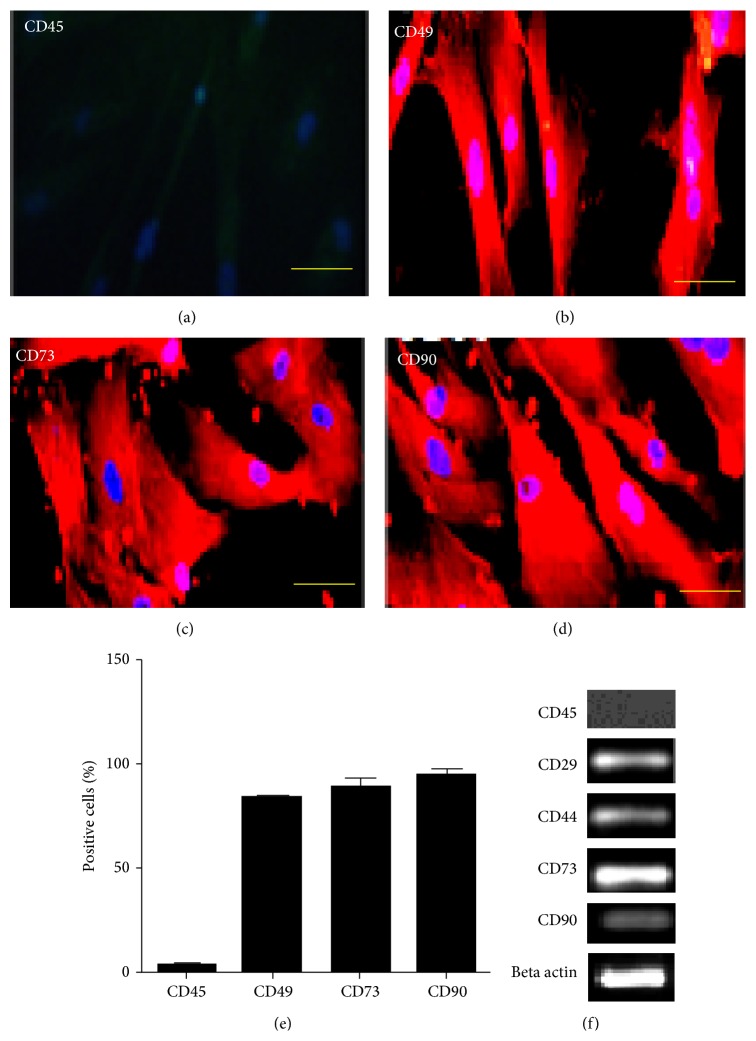
Immunostaining for UC-MSCs; (a) CD 45 negative. (b)–(d) CD49, CD73, and CD90 positive. (e) shows percentage of positive cells. RT-PCR for UC-MSCs; (f) CD29, CD44, CD73, and CD 90 positive.

AECs and UC-MSCs were characterized at passage 2 using immunostaining and RT-PCR. Both types of cells were negative for hematopoietic marker CD45 (Figures 2 and 3). AECs were positive for CK8, CK18, and CK19 using immunostaining (Figures 2(a)–2(e)) and CK16, CK18, CK19, and E-Cadherin using RT-PCR analysis (Figure 2(f)). Our results are in line with previous reports in this regard [12, 13]. UC-MSCs were positive for mesenchymal lineage markers CD49, CD73, and CD90 using immunostaining and CD29, CD44, CD73, and CD90 as indicated by RT-PCR (Figures 3(a)–3(e) and 3(f)). These results are in accordance with previously published data [6, 14–16].

### 3.3. Growth Characteristics of AECs and UC-MSCs

To determine the self-renewal ability of cells, both AECs and UC-MSCs were seeded in low numbers. Numbers of cells that form colonies were counted and plating efficiency was determined. Plating efficiency of AECs was 5.23 ± 0.72 while those of UC-MSCs were 20.41 ± 2.33 when 50 cells per cm^2^ were seeded (Table 3). A colony forming efficiency of 5.7% has been previously reported for passage 2 AECs, which is similar to plating efficiency found in our study. Similarly, plating efficiency of 12.53 ± 1.45 has been previously reported for UC-MSCs; however, this difference might be due to different number of cells plated.

We also determined maximum population doublings and doubling time for both types of cells. Number of population doublings for AECs was 7.3 ± 1.35 while those of UC-MSCs were 28.12 ± 2.5 (Table 3). It is noteworthy that proliferative potential of AECs is not much different in the initial passages compared to UC-MSCs; however, AECs proliferation declines in initial passages and they only proliferated for 4–6 passages [12]. Cord tissue UC-MSCs are highly proliferative as indicated in this study which is in accordance with previously published reports [6, 17].

### 3.4. Analysis of In Vitro Differentiation Potential

#### 3.4.1. Amniotic Epithelial Cells Can Differentiate into Keratinocytes-Like Cells

**Figure 4:**
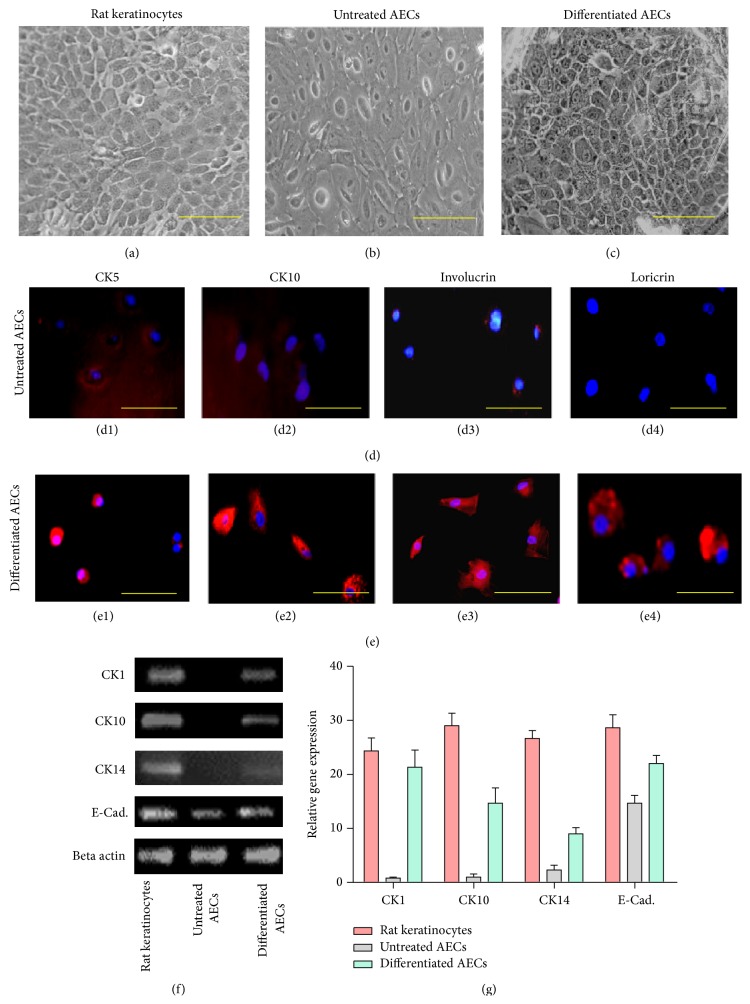
Differentiation potential of amniotic membrane epithelial cells into keratinocyte-like cells. Cytomorphology of (a) positive control, (b) untreated AECs, and (c) induced keratinocytes-like cells. Immunofluorescence staining with CK5, CK10, involucrin, and loricrin in ((d1)–(d4)) untreated AECs and in ((e1)–(e4)) induced cells. Expression of mRNA of CK1, CK10, CK14, and E-Cadherin in positive control and untreated and treated cells has been shown in (f). Expression of these genes was significantly upregulated in treated group as compared to untreated AECs. (g) showed graphical representation of RT-PCR analysis.

AECs were induced to differentiate into keratinocytes-like cells at passage 2 by culturing cells for 15 days in keratinocyte differentiation medium. Differentiation into keratinocytes-like cells was confirmed by assessing morphological changes, by immunostaining, and further by RT-PCR analysis. In the induction medium epithelial cells turned from rounded to polygonal shape, characteristic of keratinocytes (Figures 4(a)–4(c)). The immunostaining results also indicated that induced cells were positive for CK5, CK10, involucrin, and loricrin (Figures 4(d) and 4(e)) specifically expressed by keratinocytes [18]. Further, the expression of keratinocyte lineage specific genes CK1, CK10, CK14, and E-Cadherin was significantly upregulated in treated cultures as compared to control (Figures 4(f) and 4(g)). Results of this study are in accordance with other published reports that indicate upregulation of these genes in cells undergoing differentiation into keratinocytes [19].

#### 3.4.2. Differentiation of UC-MSCs into Dermal Fibroblast-Like Cells

**Figure 5:**
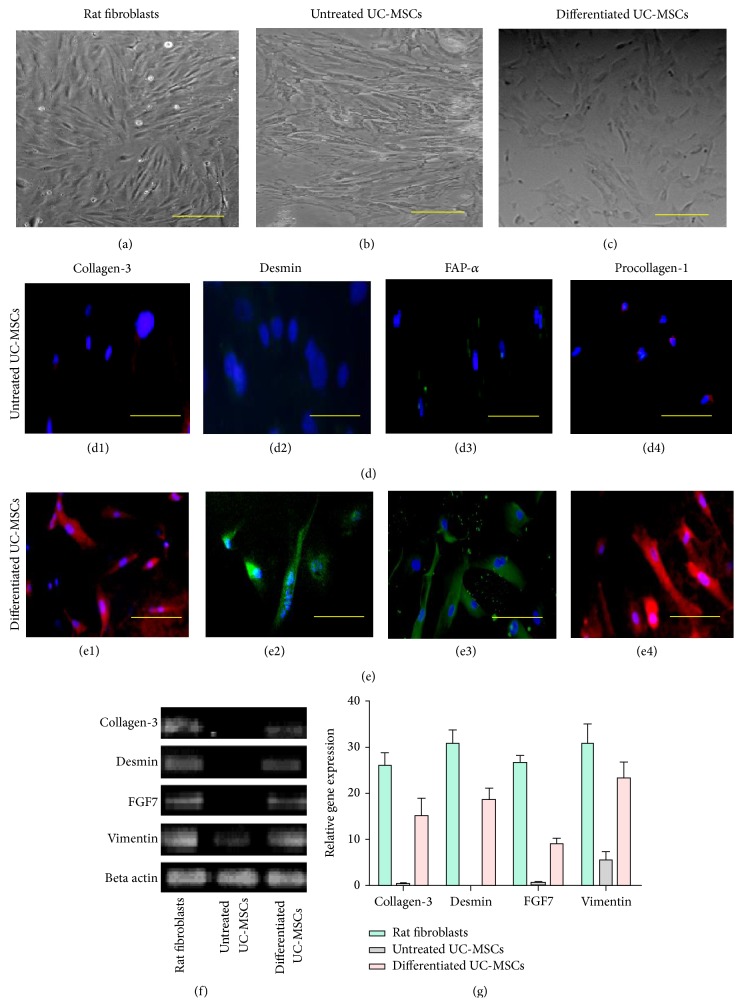
Differentiation potential of cord tissue derived UC-MSCs into dermal fibroblast-like cells. Cytomorphology of (a) positive control, (b) untreated UC-MSCs, and (c) induced dermal fibroblast-like cells. Immunofluorescence staining with collagen-3, desmin, FAP-*α*, and procollagen-1 in ((d1)–(d4)) untreated UC-MSCs and in ((e1)–(e4)) induced cells. Expression of mRNA of collagen-3, desmin, FGF7, and vimentin has been shown in (f). Expression of these genes was significantly upregulated in induced cells as compared to untreated cells. Collagen-3: collagen type 3, FGF7: fibroblast growth factor 7, FAP-*α*: fibroblast activation protein-alpha, and procollagen-1: procollagen type 1. (g) showed graphical representation of RT-PCR analysis.

Differentiation of UC-MSCs was initiated at passage 2 and experiments were terminated after 15 days. Morphology of UC-MSCs in the induction medium changed considerably (Figures 5(a)–5(c)) from fibroblastic to more irregular or triangular shape [20]. Immunofluorescence staining displayed positive expression of collagen-3, desmin, FAP-*α*, and procollagen-1 (Figures 5(d) and 5(e)). These markers are highly expressed in dermal fibroblasts [20–22]. Differentiation at the mRNA level was also observed and results indicated upregulation of collagen-3, desmin, FGF-7, and vimentin (Figures 5(f) and 5(g)).

## 4. Discussion

Parts of the placenta such as amniotic membrane and Wharton's jelly have a long history of their use in diagnostic applications. Cell populations in both amniotic membrane and Wharton's jelly are easily accessible and nontumorigenic and have ability to differentiate into variety of cell types. These characteristics have stimulated a flurry of research that aim at characterizing and evaluating their potential for use in tissue engineering and regenerative medicine. Regenerative medicine involves the use of cells to repair or replace damaged tissues for the restoration of their normal function. Stem cells are promising candidates for use in tissue engineering and regenerative medicine applications as they possess unique characteristics of self-renewal and differentiation into variety of cell types. Stem cells are present in various adult and neonatal tissues. Although adult stem cells have fewer ethical restrictions and reduced chances of teratoma formation their number, proliferation, and differentiation are limited [23, 24] making a serious limitation for use in cell-based therapy. Similarly, embryonic and induced pluripotent stem cells have problems such as legal and ethical considerations and genomic instability [25, 26]. Due to these drawbacks much attention has been paid to find alternative sources of cells for use in tissue engineering and regenerative medicine. In contrast to adult tissues, harvesting cells from neonatal sources (such as placental tissue) is a noninvasive procedure without risks to donors. Therefore, the current study was designed to evaluate the differentiation potential of human placenta derived stem cells into skin cells in an effort to appraise the potential use of these cells for the regeneration of burn injured skin.

In the present study we successfully isolated and characterized two types of stem cells from the placental tissue: epithelial cells from the amniotic membrane and UC-MSCs from cord tissue. Epithelial cells were then differentiated in vitro into the keratinocytes-like cells and UC-MSCs into dermal fibroblasts. Both enzymatic [8, 9] and nonenzymatic [6] methods have been suggested for isolation of cells from cord tissue and amniotic membrane. However, we selected nonenzymatic digestion method as it is simple, efficient, and inexpensive. Both types of cells displayed plastic adherent growth and cell outgrowth was observed 3-4 days after culturing. UC-MSCs showed characteristic fibroblast-like morphology similar to that observed with bone marrow derived MSCs [17]. The morphological features of AECs have been reported to be similar to other epithelial cells such as respiratory epithelial cells [27] with round morphology. Both UC-MSCs and AECs grew into colonies when seeded in low numbers; however, AECs raised more like a sheet of cells attached to plastic surface especially the cells from primary culture. As expected, based on previous reports, cells isolated from amniotic membrane and cord tissue showed expression of OCT4, NANOG, and SSEA4 which are stemness markers associated with embryonic origin [8–11, 28]. UC-MSCs revealed positive expression of MSC markers CD29, CD44, CD49, CD73, and CD90. Similarly, epithelial cells obtained from amniotic membrane were positive for CK8, CK16, CK18, and CK19. Both types of cells were negative for hematopoietic marker, CD45, as determined by immunostaining and RT-PCR. This analysis showed that there was no contamination of hematopoietic stem cells from cord blood.

Previously, we and others have shown that UC-MSCs derived from cord tissue have multilineage differentiation potential. In the current study, AECs expressed morphology of keratinocytes after in vitro induction. Immunostaining results indicated that differentiated cells were positive for the expression of CK5, CK10, involucrin, and loricrin. Differentiation into keratinocyte-like cells was further confirmed by significantly upregulated mRNA levels of CK1, CK10, CK14, and E-Cad. genes observed in induced cultures compared to controls.

UC-MSCs were induced to differentiate into dermal fibroblasts at passage 2. In the induction medium, differentiated cells showed changes in their morphology. Moreover, immunostaining of induced cells showed strongly positive expression of collagen-3, desmin, FAP-*α*, and procollagen. Similarly, upregulation of desmin, FGF7, and vimentin as measured with quantitative PCR further confirmed differentiation of UC-MSCs into dermal fibroblasts. These findings indicate that UC-MSCs could be induced to differentiate into dermal fibroblasts in vitro and could be used as seed cells for reconstructing off the shelf skin products to be used for skin engineering.

## 5. Conclusion

In conclusion, AECs and UC-MSCs can be induced to differentiate into keratinocytes and fibroblasts in vitro.

## Figures and Tables

**Table 1:** List of used primers and their sequences.

Number	Markers	5′-3′ sequence	Product size
1	Beta actin	CGCATGGGTCAGAAGGATTC (F)	137
		TAGAAGGTGTGGTGCCAGATTT (R)	

2	CD29	GCAGTTGGTTTTGCGATTAAG (F)	233
		AAGGCATCACAGTCTTTTCCA (R)	

3	CD44	AGAAAAATGGTCGCTACAGCA (F)	571
		CTGAAGTGCTGCTCCTTTCAC (R)	

4	CD45	CACTGCAGGGATGGATCTCA (F)	312
		ACTCGTGGGTTCAGAACCTTCA (R)	

5	CD73	ACAACAGCCAACTGCTTTCAT (F)	154
		TTCTCAGCATTCCCGAAAT (R)	

6	CD90	ATGAACCTGGCCATCAGCATGC (F)	344
		CACGAGGTGTTCTGAGCCAGCA (R)	

7	CK1	GGAGGAGGAGGTGGTAGATTTT (F)	388
		GAGGTTGCTGATGTATGACTCG (R)	

8	CK10	GAGCAAGGAACTGACTACAG (F)	249
		CTCGGTTTCAGCTGCAATCT (R)	

9	CK14	TGCTATTGGTGTCAGGGAAG (F)	277
		GTGGCAAGGTTCTTTTCTCC (R)	

10	CK16	ATCGTTAGAGCCAAGCAGGA (F)	228
		GAGAAGCGAGAGGGAGGTGAC (R)	

11	CK18	CACACTCACGGAGCTGAGAC (F)	168
		GCCAGCTCTGACTCCAGATG (R)	

12	CK19	GCCTGGCTGCAGATGACT (F)	157
		AGCTCCTCCTTCAGGCTCTC (R)	

13	Collagen-3	GTTGACCCTAACCAAGGATGCA (F)	203
		GGAAGTTCAGGATTGCCGTAG (R)	

14	Vimentin	CTGCGGGAGTAGTTGGAAAGT (F)	241
		GGAAATGGGACAAAACATCCT (R)	

15	FGF 7	TGGTGAAGTTCATGGATGTCTATC (F)	212
		CACAGGATGGCTTGAAGATGTA (R)	

16	Desmin	CATCCTCAAGAAGGTGTTGGAG (F)	112
		CAAAGAGACGTGGGACGAGT (R)	

17	Oct-4	GGCGTTCTCTTTGGAAAGGTGTTC (F)	145
		CTCGAACCACATCCTTCTCT (R)	

18	Nanog	GGACGCGTGGGGGCTGGAGAC\ (F)	174
		GGCTCGAGGGGGACCAGGAAG (R)	

19	SSEA4	CCGCGTCAAGAGGCCCATGAA (F)	148
		CCCGCTTCTCGGTCTCGGACAA (R)	

**Table 2:** List of reagents and antibodies.

Sr. number	Reagents/antibodies	Details
1	Phosphate buffered saline	Sigma Aldrich, USA
2	Fetal calf serum	Sigma Aldrich, USA
3	Penicillin and streptomycin	Sigma Aldrich, USA
4	DMEM	Sigma Aldrich, USA
5	0.25% trypsin-EDTA	Sigma Aldrich, USA
6	Crystal violet dye	Sigma Aldrich, USA
7	Fetal bovine serum	Sigma Aldrich, USA
8	HAM F12	Sigma Aldrich, USA
9	Hydrocortisone	Sigma Aldrich, USA
10	Insulin transferrin	Sigma Aldrich, USA
11	DAPI	Sigma Aldrich, USA
12	CK5 antibody	Sigma Aldrich, USA
13	CK10 antibody	Sigma Aldrich, USA
14	Involucrin antibody	Sigma Aldrich, USA
15	Insulin	Sigma Aldrich, USA
16	Basic fibroblast growth factor	Sigma Aldrich, USA
17	Trizol reagent	Sigma Aldrich, USA
18	Reverse transcriptase kit	Invitrogen, USA
19	Keratinocytes growth factor	Invitrogen, USA
20	CD45 antibody	Abcam, UK
21	CK8 antibody	Abcam, UK
22	CK18 antibody	Abcam, UK
23	CK19 antibody	Abcam, UK
24	CD49 antibody	Abcam, UK
25	CD73 antibody	Abcam, UK
26	CD90 antibody	Abcam, UK
27	FAP-*α* antibody	Abcam, UK
28	Loricrin antibody	Abcam, UK
29	Collagen-3 antibody	Santa Cruz Biotechnology, USA
30	Desmin antibody	Santa Cruz Biotechnology, USA
31	E-Cadherin	Santa Cruz Biotechnology, USA
32	Procollagen-1 antibody	Santa Cruz Biotechnology, USA

**Table 3:** Growth characteristics of AECs and UC-MSCs as determined by using parameters such as PDs, PDT, and PE. PDs: population doublings, PDT: population doubling time, and PE: plating efficiency.

Biological samples	PDs	PDT (hours)	PE (%)
AECs	7.3 ± 1.34	140 ± 20	5.23 ± 0.72
MSCs	28.12 ± 2.5	50 ± 6.0	20.4 ± 2.33

## Abbreviations

AECs: Amniotic epithelial cells
UC-MSCs: Umbilical cord mesenchymal stem cells
CK: Cytokeratin
DMEM: Dulbecco's modified eagle medium.
